# A hybrid TCN-GRU model for classifying human activities using smartphone inertial signals

**DOI:** 10.1371/journal.pone.0304655

**Published:** 2024-08-13

**Authors:** Sarmela Raja Sekaran, Ying Han Pang, Lim Zheng You, Ooi Shih Yin

**Affiliations:** Faculty of Information Science and Technology, Multimedia University, Malacca, Malaysia; Air University, PAKISTAN

## Abstract

Recognising human activities using smart devices has led to countless inventions in various domains like healthcare, security, sports, etc. Sensor-based human activity recognition (HAR), especially smartphone-based HAR, has become popular among the research community due to lightweight computation and user privacy protection. Deep learning models are the most preferred solutions in developing smartphone-based HAR as they can automatically capture salient and distinctive features from input signals and classify them into respective activity classes. However, in most cases, the architecture of these models needs to be deep and complex for better classification performance. Furthermore, training these models requires extensive computational resources. Hence, this research proposes a hybrid lightweight model that integrates an enhanced Temporal Convolutional Network (TCN) with Gated Recurrent Unit (GRU) layers for salient spatiotemporal feature extraction without tedious manual feature extraction. Essentially, dilations are incorporated into each convolutional kernel in the TCN-GRU model to extend the kernel’s field of view without imposing additional model parameters. Moreover, fewer short filters are applied for each convolutional layer to alleviate excess parameters. Despite reducing computational cost, the proposed model utilises dilations, residual connections, and GRU layers for longer-term time dependency modelling by retaining longer implicit features of the input inertial sequences throughout training to provide sufficient information for future prediction. The performance of the TCN-GRU model is verified on two benchmark smartphone-based HAR databases, i.e., UCI HAR and UniMiB SHAR. The model attains promising accuracy in recognising human activities with 97.25% on UCI HAR and 93.51% on UniMiB SHAR. Since the current study exclusively works on the inertial signals captured by smartphones, future studies will explore the generalisation of the proposed TCN-GRU across diverse datasets, including various sensor types, to ensure its adaptability across different applications.

## 1 Introduction

In recent years, data scientists have been keen to delve into the concept of human activity recognition (HAR) due to its potential across a wide range of applications, including geriatric/patient monitoring, personal healthcare, remote monitoring, assisted ambient living, sports, environmental monitoring, security surveillance, etc. Adopting HAR systems in these fields helps improve the people’s quality of life. For example, one can track and keep up with their physical fitness and avoid being sedentary using an automatic physical activity monitoring system.

HAR systems recognise/predict the motion performed by an individual based on the information gathered from different sensors. Cameras, wearable sensors (i.e., accelerometers and gyroscopes), smartphones and smartwatches are the widely used sensors in the HAR domain. Without efficient and effective HAR systems, most fields, as mentioned above, will require strenuous labour and constant monitoring from the labourers. For instance, geriatric caretakers work around the clock to monitor and track their patient’s activities to prevent fall accidents. Hence, developing a robust HAR system is essential as it can help reduce labour and provide accurate activity tracking without human intervention.

HAR systems are generally categorised into two broad classes, namely computer vision-based HAR and sensor-based HAR. The main distinguishing factor between these categories is the type of input the recognition system receives. For example, computer vision-based HAR receives two/three-dimensional inputs (i.e., images or videos) from the camera and processes them for classification. In contrast, sensor-based HAR takes in one-dimensional (1D) inertial signals from sensors like accelerometers, gyroscopes, smartphones, and smartwatches. This research work focuses on developing a sensor-based HAR, specifically a smartphone-based HAR. Sensor-based HAR was chosen in this work as it has several advantages over computer vision-based HAR, and the advantages are as follows:

- Sensor-based HAR requires relatively less computational resources to pre-process and retain 1D inertial signals.
- Unlike vision-based HAR, the performances of sensor-based HAR systems are not susceptible to external factors like irrelevant activities (i.e., the motion of inanimate objects and animals, etc.), varying illumination and occlusions [1].
- Sensor-based HAR protects user privacy since no user identification information is collected nor revealed, whereas vision-based HAR violates user privacy as the individual’s identification attributes, like face data, are captured during data collection [2].

**Fig 1:**
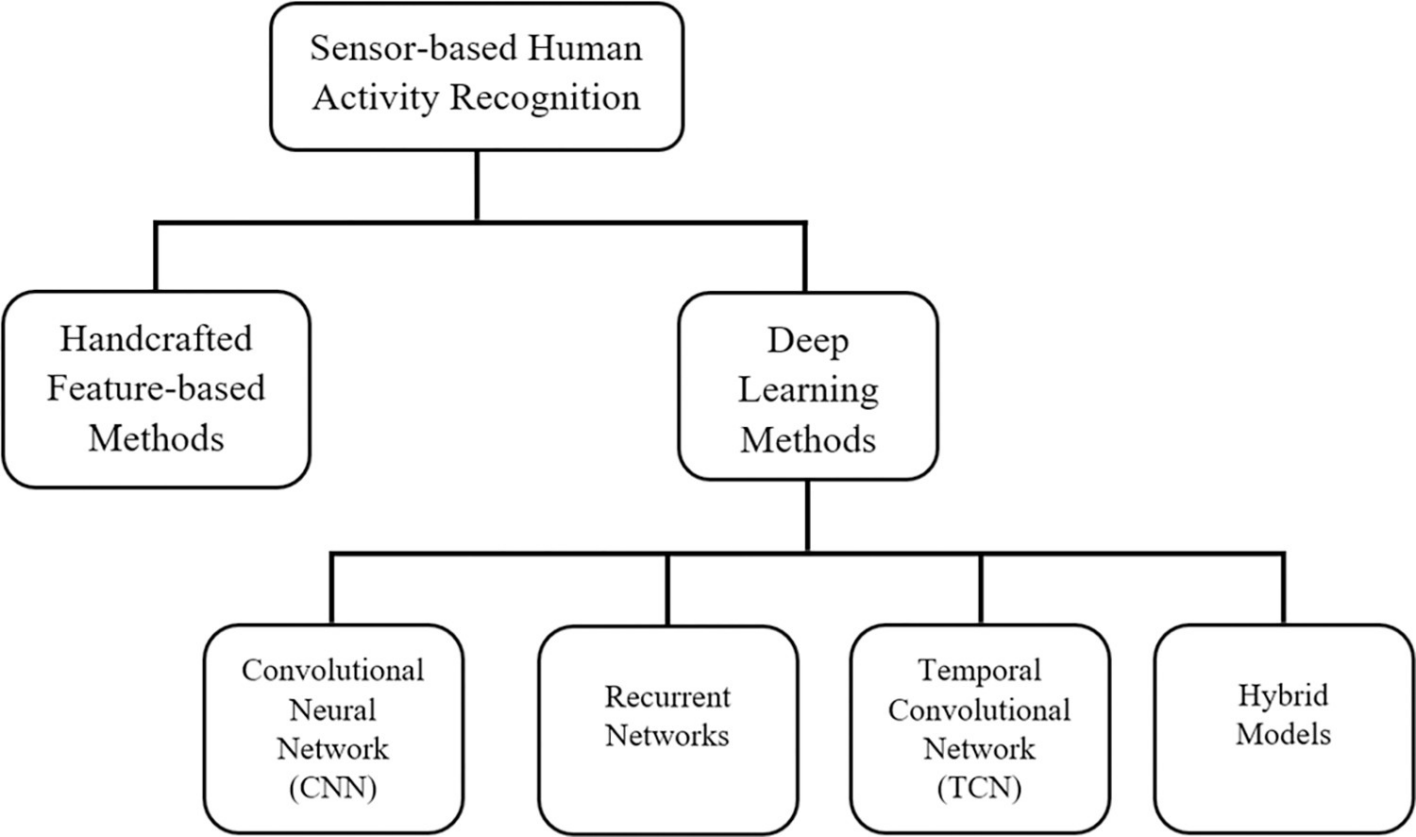
Sensor-based human activity classification techniques.

Owing to this, researchers have proposed numerous sensor-based HAR models throughout the years to recognise human activities. Fig 1 shows the existing HAR solutions. In the past few decades, handcrafted feature-based methods were generally preferred over deep learning methods due to insufficient computational resources. In a handcrafted feature-based approach, data scientists thoroughly study and analyse motion data to understand its nature and characteristics. Then, they develop feature engineering and extraction techniques to attain the desired features that can distinguish human activities effectively. However, researchers have shifted their attention towards deep learning methods due to the exponential growth of computing power. The fundamental reason behind this shift is that deep learning models automatically capture desired patterns/features from the input sequences without demanding human supervision and accurately predict unseen future events. Convolutional Neural Networks (CNN) [3–6], recurrent networks [7–9], Temporal Convolutional Networks (TCN) [10–13] and hybrid models [14–16] are widely used deep learning architectures in this domain.

### 1.1 Related work

As mentioned earlier, various solutions, including handcrafted feature-based and deep learning methods, have been proposed for HAR. The following subsections present existing literary works on handcrafted feature-based and deep learning methods.

#### 1.1.1 Handcrafted feature-based methods

In earlier years, the researchers favoured handcrafted feature-based techniques for human motion recognition due to limited computational resources. Researchers scrutinised the inertial signals to understand the characteristics and behaviours of the signals. With prior domain knowledge, the researchers were able to construct feature engineering techniques to capture underlying patterns in the time and/or frequency domain that describe the nature of human activity for classification. Next, the extracted features might undergo feature ranking and selection processes to ensure essential features were chosen, and redundant information was removed. Finally, these features were classified into respective activity classes using traditional machine learning models, namely Support Vector Machine (SVM), Random Forest (RF), K-Nearest Neighbour (KNN), Hidden Markov Model (HMM), boosting algorithms, etc.

Anguita et al. performed a series of statistical feature calculations (e.g., mean, standard deviation, signal entropy, interquartile range, median absolute value, etc.) on the input motion data to compute 561 features [17]. The authors employed a multi-class SVM model to classify the generated statistical features. Similarly, Garcia-Gonzalez et al. computed time-dependent statistical features using inertial signals before passing to a multi-class SVM model equipped with a Radial Basis Function (RBF) kernel for activity recognition [11]. In the work of [18], the authors extracted six different statistical features in the frequency domain and employed a multi-class SVM with Particle Swamp Optimisation as a classifier.

Several authors have shown their preference for RF classifiers over other machine learning algorithms due to the superior recognition performance of the RF classifiers. For instance, Kee et al. adopted the RF classifier for data classification to study the effects of multiple feature selection techniques, including Correlation-based Feature Selection Subset evaluator (CFS), Correlation-based Attribute (CA) Evaluator, and Information Gain (IG) Attribute Evaluator [19]. Vijayvargiya et al. extracted five different time-dependent features (i.e., variance, mean absolute value, skewness, kurtosis and root mean square) from human motion signals for activity classification [20]. The authors trained and tested these features on different machine learning models. The empirical results exhibited that the RF model achieved a higher accuracy than the other models by achieving 92.71%.

Besides, Mohsen et al. proposed a KNN model with 20 neighbours to classify human activities [21]. The findings indicated that a higher number of neighbours was able to reduce noise and improve accuracy. Liu et al. proposed an enhanced KNN variant, coined as Reduced Kernel KNN (RK-KNN) [22]. The authors incorporated kernel methods into the KNN model to obtain high-dimensional features for enhanced classification performance. However, processing high-dimensional features might increase the computational load. Thus, the authors applied a reduced kernel technique to improve computational efficiency.

The boosting algorithm is another popular handcrafted feature-based approach. It is widely used in data classification for its capability to reduce bias by combining multiple weak learning models for final predictions. Kataria et al. applied multiple machine learning algorithms to human motion classification, with the Extreme Gradient Boosting (XGB) model demonstrating superior performance compared to the other classifiers [23]. In the study, the authors further improved the classifier’s performance by implementing the Grid Search technique to determine optimal hyperparameters. On the other hand, Wang et al. designed a real-time walking motion detection system based on two algorithms: a copula-based outlier detection (COPOD) and an enhanced light gradient boosting machine (LightGBM) model [24]. Firstly, the authors amalgamated focal loss, an improved cross-entropy loss function specifically for class imbalance problems, with LightGBM to overcome class imbalance during multi-label classifications. Additionally, the authors put forward a novel denoise method by fusing the Window matrix with Outlier Detection (W-OD) to minimize the noise effects on the model performance without altering any input data.

Although most handcrafted feature-based methods yield adequate performance, these methods have several drawbacks, as follows:

- Complex pre-processing and manual feature engineering are required on the input inertial data for satisfactory classification performance.
- Handcrafted feature-based methods require feature extraction, ranking, and selection processes, which are tedious and time-consuming [25, 26].
- Manual feature engineering techniques are highly dependent on prior knowledge and may overlook the underlying implicit patterns of the inertial signal that are significant for distinguishing between activities. Furthermore, classification performance might degrade when handling unseen subjects due to lacking generalisability [27].
- No universally accepted standards for feature ranking and selection methods exist, as they differ between studies [28].

#### 1.1.2 Deep learning methods

In light of technological advancements, the availability of cheap, faster, and more efficient computing devices has increased exponentially. This growth has broken the technological barrier and nudged countless researchers to explore deep learning algorithms. As a result, numerous ground-breaking deep learning models have been developed lately. Deep learning models are capable of automatic learning/extraction of desired features, resulting in a high-performing model. CNN, recurrent models, TCN, hybrid models, etc., are the leading deep learning models in most domains, and these models have been adopted into the HAR domain throughout the years for effective activity recognition.

Yazdanbakhsh et al. developed a CNN model with alternating strided and dilated convolutions for activity classification [3]. Instead of directly processing the raw motion signals, the authors transformed the motion signals into image modules and classified them for better performance. Tang et al. introduced Lego filters and embedded them into the CNN model for memory and computation reduction [5]. These filters operate in lower dimensions, alleviating the overall model parameters and leading to lightweight computation. Khan et al. introduced a CNN network consisting of multiple convolutional heads and an attention module for motion classification [4]. This model extracts features from inertial signals at different scales for better learning. The authors adopted the squeeze and excitation module (SE) to enhance the salient features and remove the redundant ones.

However, Asim et al. argued that conventional CNNs may be suboptimal for time series classification, especially in the HAR domain, since the models struggle to learn temporal features which are significant for gait analysis [29]. Hence, recurrent networks are alternative optimal architectures to classify time series data due to their competency in extracting temporal features. Since Recurrent Neural Networks (RNN) are susceptible to short-term memory problems where the gradient tends to either explode or vanish during training [30], less adoption has been done in human gait recognition. Unlike RNN, Long Short-Term Memory (LSTM) architectures are extensively implemented in motion signal analysis tasks because they are resistant to vanishing and exploding gradient effects. Pienaar et al. devised a recurrent model with three LSTM layers and applied L2 regularisation to reduce model overfitting [9]. Likewise, Ullah et al. proposed a stacked LSTM network comprising five LSTM layers for extracting time-dependent features and a softmax output layer for classification [8]. The authors also implemented L2 regularisation to leverage its benefits. Hernandez et al. introduced bidirectional LSTM, where the information flows both ways to prevent information loss [31]. Despite the LSTM model’s performance, these models require heavy computations to capture salient features and provide accurate predictions.

TCN models are specifically designed for time series classifications (i.e., weather prediction [32], speech recognition [33], etc.) because these models are relatively lightweight in computation and can preserve longer-term dependency throughout the model. Lea et al. introduced new TCN architectures, coined as Dilated TCN and Encoder-Decoder TCN, for their action segmentation work [34]. Nair et al. utilised these models to classify human activities [10]. The authors tuned both models using inertial data to improve recognition accuracy. Furthermore, Garcia et al. introduced two different TCN variants, TCN-FullyConnectedNetwork (TCN-FCN) and deepConvTCN, and evaluated them on PAMAP2 and OPPORTUNITY datasets [35]. These models achieved high accuracy on both datasets. Besides, Raja Sekaran et al. proposed a variant of TCN coined as Multiscale Temporal Convolutional Network (MSTCN), which is based on the Inception architecture [12]. This model extracts features from the input sequences at different scales, capturing complex temporal patterns. Although MSTCN has shown high performance in human activity recognition, it is deep and complex, containing 3.75 million trainable parameters. The overall computational complexity is relatively high.

Another popular approach in the HAR domain is the hybrid model. A hybrid model combines two or more different deep learning models into one model to perform activity classification. For example, Mutegeki et al. integrated a CNN and LSTM architecture to develop a hybrid model for deep spatiotemporal feature extractions [14]. The authors reported that their CNN-LSTM is better at classifying human motion than the conventional LSTM. Similarly, Andrade-Ambriz et al. built a CNN-LSTM architecture for motion recognition using three-dimensional input data (e.g. videos) [16]. Besides achieving high classification, the author stated that their model only needs a relatively short time for training and inference. Xu et al. introduced a hybrid model known as InnoHAR [15]. InnoHAR is comprised of a CNN architecture inspired by the Inception Model and followed by a GRU network. The authors adopted Inception Network as it allows multiscale feature extraction. InnoHAR demonstrated a promising recognition performance during model evaluation.

Additionally, researchers have directed their efforts towards building deep ensemble learning models for human activity classification. These models integrate different algorithms and leverage the architectural benefits of these diverse algorithms for better final predictions. For instance, Tan et al. proposed an ensemble learning algorithm (ELA) by employing GRU, CNN and deep neural network (DNN) [36]. CNN-GRU, GRU and DNN are utilised for feature extraction in such a way that CNN-GRU, GRU and DNN analyse the original input signals, the reshaped signals and the computed statistical features, respectively. After that, the generated features are fed into DNN for final classification. The proposed ELA model exhibited a promising performance with an accuracy of 96.7% on the UCI HAR dataset, outperforming other methods for human activity classification. Similarly, Bhattacharya et al. proposed Ensem-HAR model by constructing three different models: CNN-net, CNNLSTM-net, ConvLSTM-net, and StackedLSTM-net using convolutional layers and LSTM units. The authors gathered the predictions from these three models and combined them to create a new meta-learner training set. The Random Forest classifier was chosen as the meta-learner due to its superior performance.

### 1.2 Motivation and contribution

Over the past decades, innumerable solutions have been proposed for HAR systems. However, as discussed in the earlier subsection, several challenges still obstruct the potential model performance. In summary, the challenges in developing an effective smartphone-based HAR model are:

- Most HAR models that require time-consuming manual feature extraction and selection suffer from a lack of model generalisation due to the heavy dependence on prior knowledge [27]. Moreover, the lack of universal standards for choosing optimal features has made HAR modelling difficult [28].
- The conventional CNN models enable automatic spatial feature extraction, which is essential for image/video-based classification tasks. However, these models hardly capture temporal information, which is significant in analysing human motion signals, as these features better represent underlying patterns of the activities [29].
- LSTM networks are known for time series classification because these networks can capture relatively longer temporal features to provide richer information for classification. Nevertheless, these models need high computational power and memory during training as the input signals flow through multiple gate operations and the intermediate results are stored in the network [37].
- TCN models require relatively low computation and have the ability to preserve longer-term dependency of the inertial signals in the network [37]. However, these models must be denser by stacking convolutional layers with many large-sized filters to achieve satisfactory results [10, 35].

**Fig 2:**
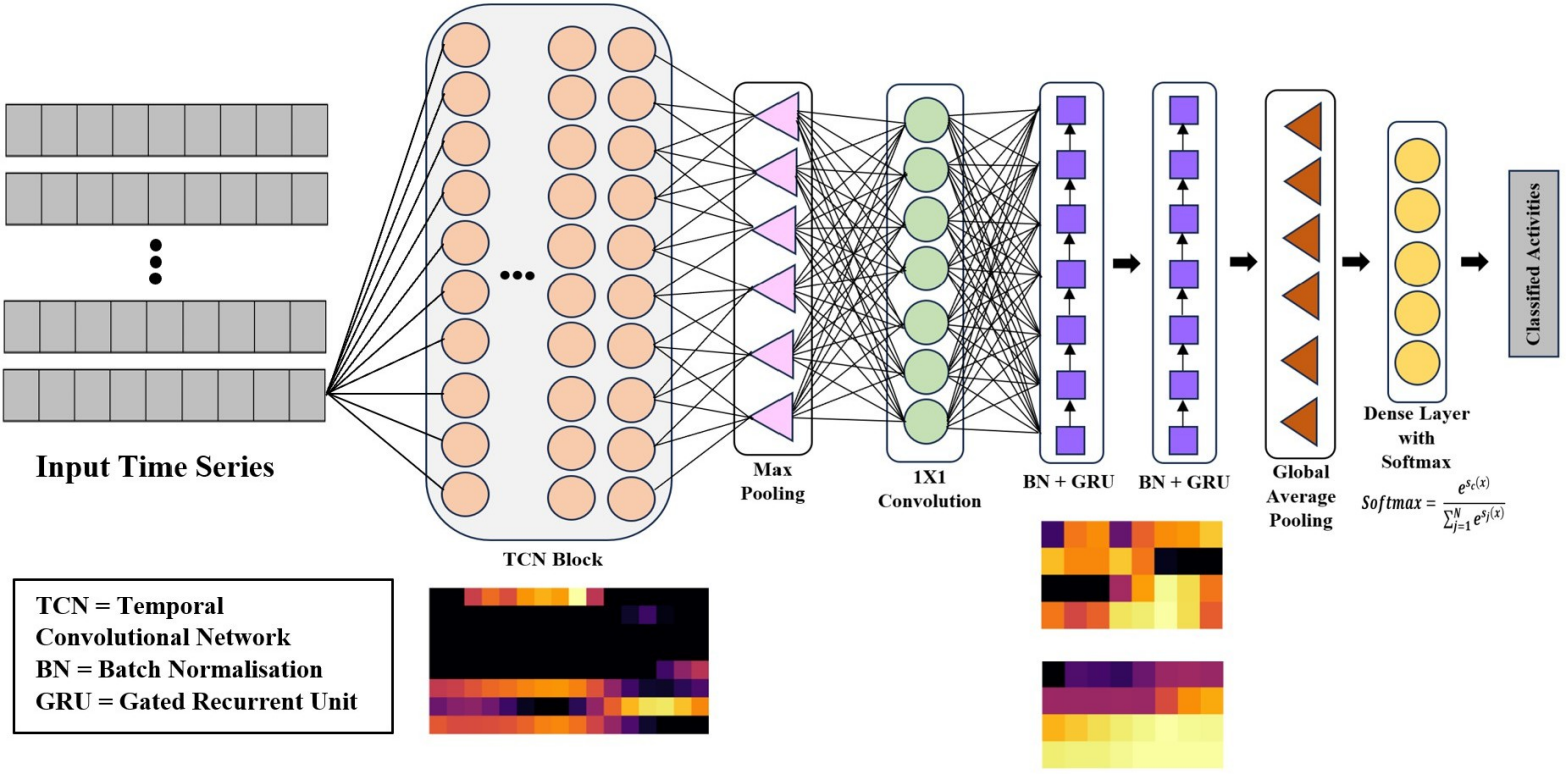
Architecture of TCN-GRU model.

Considering the above-mentioned limitations, we devise a hybrid deep learning architecture integrating a TCN architecture with a Gated Recurrent Unit (GRU), coined as TCN-GRU, for smartphone-based human activity recognition. Fig 2 illustrates the overview of the proposed TCN-GRU. The contributions of this work are as follows:

- By employing stacks of one-dimensional dilated convolutions and GRU layers, TCN-GRU can extract spatiotemporal features from the input inertial signals without any time-consuming manual feature engineering or feature selection techniques.
- TCN-GRU is a lightweight deep learning model that achieves promising accuracies with only ~0.18 million (M) parameters. Besides, it has a relatively short training period. This is because the model architecture is equipped with fewer short filters and dilations to reduce the overall parameters without negatively affecting the model performance.
- Integrating dilations, residual connections, and GRU layers helps the model preserve longer-term dependency by retaining longer past information of the inertial signals.
- Comprehensive system performance evaluation is conducted on two different smartphone-based HAR datasets: UCI HAR and UniMiB SHAR. Subject-independent and dependent testing protocols are examined to analyse the system’s robustness across different testing scenarios.

## 2 Proposed method

The proposed TCN-GRU model is composed of several components, including TCN block, Max pooling (MaxPool), GRU layers, and Global Average Pooling (GAP). The integrated architecture leverages the strengths of each component for an enhanced classification performance. Each component is explained in the subsequent subsections.

### 2.1 Temporal Convolutional Network (TCN)

As previously mentioned, TCN models are implemented in a variety of time series recognition applications due to their ability to expedite more efficient classification. These models can take up any input sequence length and produce a same-length output sequence. Besides, TCN models can perform convolutional operations parallelly by applying the same filters in each layer, leading to a highly parallelisable model [37]. Moreover, integrating different dilations in the convolutional layers allows flexible receptive field sizes. The receptive field is the kernel’s visible region of the input sequence during convolutional operations. The dilations enlarge the receptive field of the convolutional kernels according to the applied dilation rate, allowing longer time-dependent feature extraction without raising the model parameters excessively [38]. Furthermore, TCN components, such as dilated causal convolutions and residual connections, retain extensive past information about the motion signals throughout the model. The stability of the gradients is also preserved during model training since the residual connections make the models less vulnerable to the vanishing and exploding gradient problems [39]. Lastly, these models demand relatively lower computational power and memory resources during training because they do not implement gate operation and have no partial results to store.

Owing to this, we adapt the TCN architecture into the proposed model. Inspired by the original Dilated TCN model proposed by Lea et al. [34], we further enhance the TCN model to boost its performance. The proposed TCN differs from the original Dilated TCN in several ways, as follows:

- Lea et al. implemented acausal convolutions in the original model. In contrast, our enhanced TCN variant utilises causal convolutions as it prevents future information from leaking into the past by preserving the length and order of the input signals [40].
- Unlike the original Dilated TCN, dropout is adopted instead of spatial dropout.
- The proposed TCN implements batch normalisation instead of channel normalisation.
- ReLU activation is chosen instead of wavenet activation in the proposed TCN.

**Fig 3:**
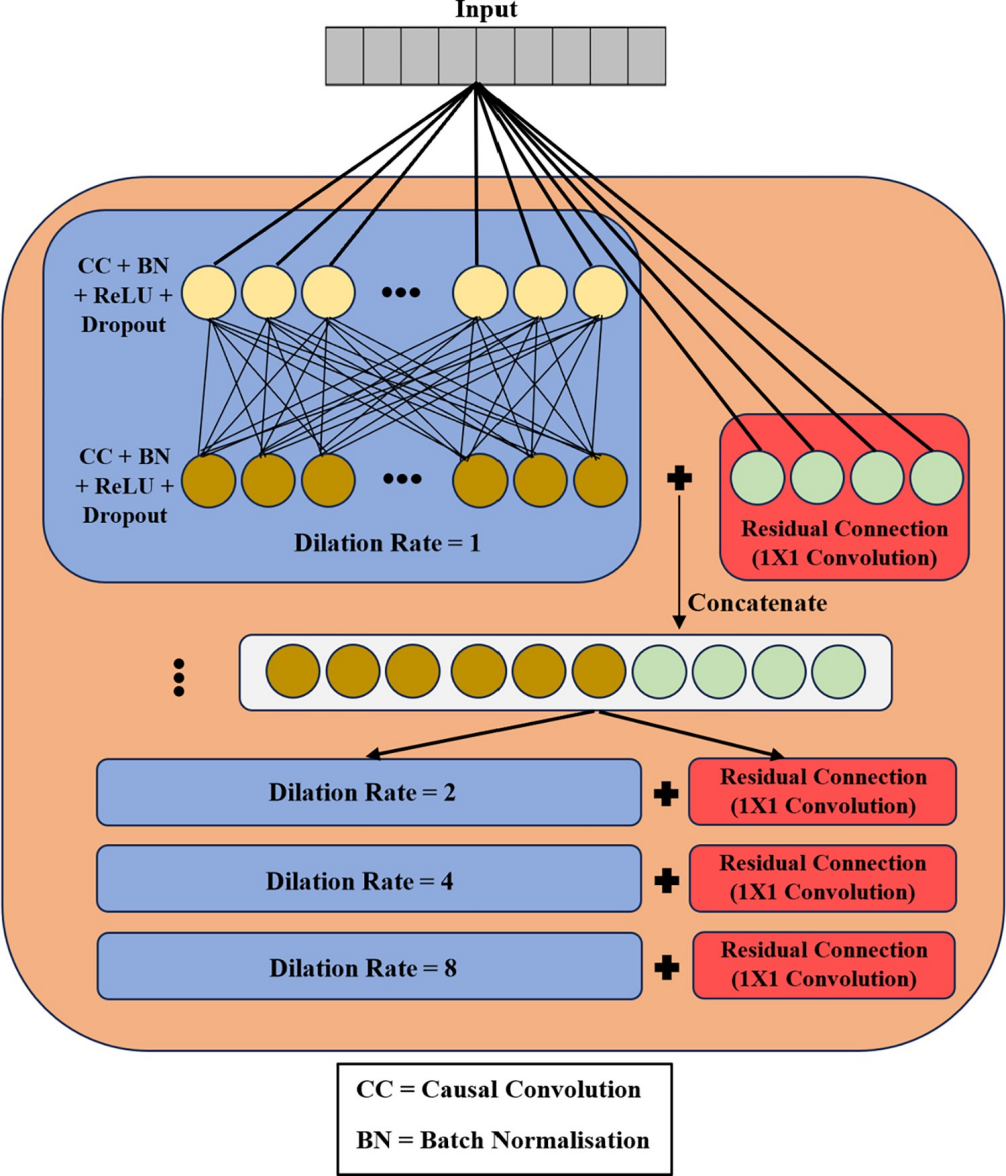
Architecture of the enhanced TCN model.

Fig 3 illustrates the detailed framework of the enhanced TCN variant. The TCN model contains dilated causal convolutional layers, batch normalisation layer, ReLU activation, dropout layer, and residual connection. TCN networks can accommodate multiple TCN blocks. However, in the proposed TCN-GRU model, only one TCN block is utilised for minimal overall parameters. In a TCN block, there are multiple dilation levels with varying dilation rates, *d*. Each dilation level consists of two dilated causal convolutions, each followed by a batch normalisation layer, a dropout layer and a ReLU activation layer. Parallelly, there is a residual connection with a one-by-one convolutional layer in every dilation layer, and the residual connection’s output is concatenated with the dropout layer’s output.

**Fig 4:**
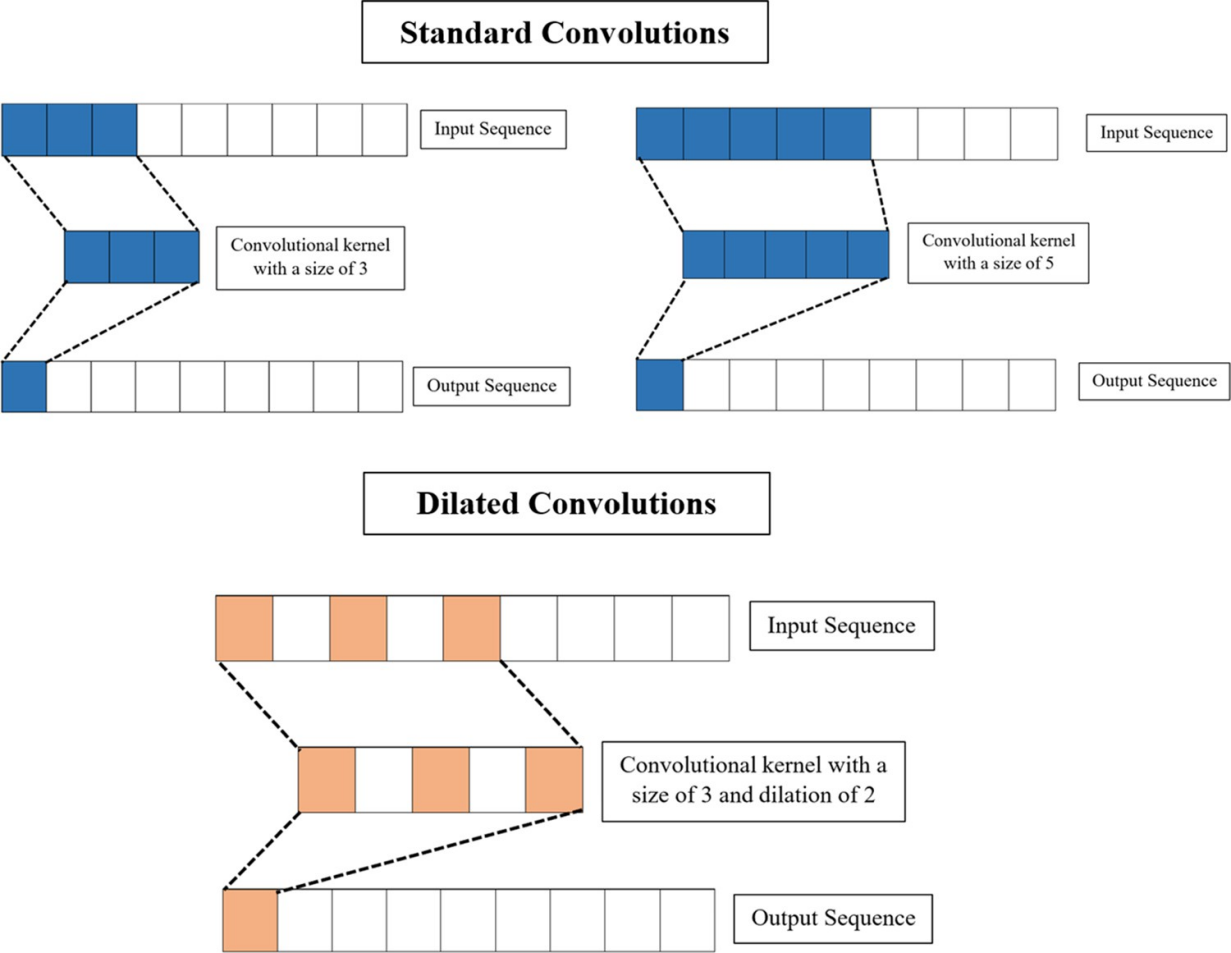
Difference between standard and dilated convolution.

Dilated causal convolutional layers are selected over conventional ones due to their efficiency in analysing the inertial data. Fig 4 illustrates the conventional convolutional operation and dilated convolutional operation. The conventional convolutional layers require longer kernels to extract long temporal features. This increases the overall parameters and computational complexity. In contrast, dilated causal convolutional layers utilise dilations to improve the model’s classification performance while reducing the model’s complexity. These convolutional kernels are expanded by inserting zeros between the values. Thus, the receptive fields of the convolutional kernels are widened, facilitating the capture of longer temporal features without increasing model parameters. Additionally, dilated convolutional kernels are equipped with causal paddings to prevent the model from violating the input’s sequence order.

Another vital component in the enhanced TCN is the residual connections. Adding residual connections to a model provides an alternative path for the gradients to flow throughout the model during training without passing through any non-linear activation functions. Hence, the model is able to maintain stable gradient flow during training, where the gradients do not either vanish or explode during backpropagation. Consequently, stable gradient flow ensures the model sustains a longer-term dependency of the input motion signals throughout the training [7]. The residual connection in the proposed TCN-GRU includes a one-by-one convolutional layer. This layer generates outputs of the same size as the dropout layer’s output so that the subsequent concatenation layer receives the same-sized inputs.

The most common issue encountered while training a deep learning model is the internal covariate shift problem, where every layer’s input distribution changes continuously as the earlier layers’ parameters change. This can result in the model converging slower during the training [41]. Hence, a batch normalisation layer is employed after each dilated causal convolutional layer to resolve the internal covariate shift problem. The batch normalisation layer introduces two new trainable parameters, updated during the model training. In batch normalisation, mean and variance are calculated for each layer distribution, and the output vector is normalised using those values. The optimal values for two learnable parameters are determined during training and used to scale and shift the normalised output vector [41].

Additionally, ReLU activation is integrated into the proposed TCN-GRU to introduce non-linearity. There are several benefits to implementing ReLU activation. Firstly, ReLU activations are computationally simple since they do not require exponential computation during training [42]. Furthermore, the output of the ReLU activation ranges from zero to infinity, with all the negative values being converted into zeros. Therefore, ReLU activation can produce an output with true zero values, generating multiple zeros in the activations of the model’s layers. This phenomenon increases the network’s sparsity representation, simplifying the model and accelerating the training process [43]. Moreover, ReLU activation also resolves the vanishing gradient problem [44].

Generally, deep learning models, especially if training samples are too small or dense networks with huge parameters, are prone to overfitting problems [15]. Overfitting is a problem where the model has a good classification accuracy with training samples, but the performance deteriorates when tested with unseen new data. Thus, regularisation is vital to any deep learning network to reduce model overfitting. We implemented dropout layers with a rate of 0.05 in our proposed TCN-GRU model. The dropout layer randomly ignores the neurons in the model’s layers according to the pre-set dropout rate. As a result, only certain neuron weights are updated during training, increasing the decorrelation between neuron weights throughout the model. Additionally, the dropout regularisation increases the sparsity of the model, allowing better model generalisation.

The extracted features from the TCN blocks are fed into a max pooling layer before being passed into the GRU layers. Max pooling is an operation where a max filter is applied over the non-overlapping subregion of the feature maps to produce an intermediate representation. Implementing max pooling can reduce input dimensionality, thereby minimising the number of model parameters and reducing the computational costs. Moreover, max pooling can reduce the overfitting effect and eliminate noise from the input sequences [45]. After that, the output of max pooling layers undergoes a convolutional operation in a one-by-one convolutional layer.

### 2.2 Gated Recurrent Network (GRU)

**Fig 5:**
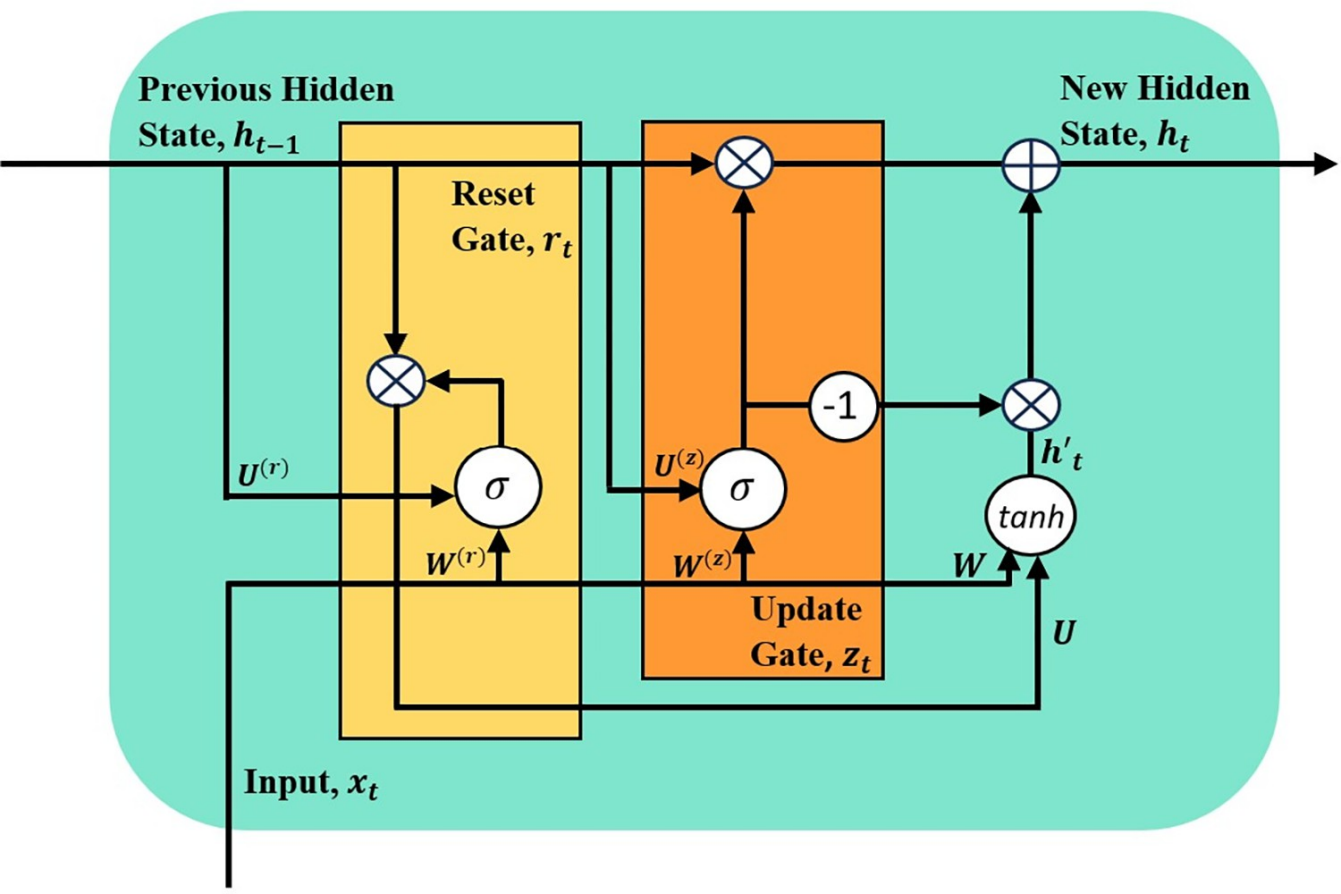
Architecture of the GRU layer.

Cho et al. developed a recurrent model known as Gated Recurrent Unit (GRU) that resolves a prevalent problem in the conventional Recurrent Neural Network (RNN), which is the vanishing and exploding gradient during model training [46]. Fig 5 illustrates the architecture of the standard GRU layer. The GRU network offers certain advantages over the LSTM network. One significant advantage is that it is a relatively simple model with reduced parameters and requires lower resources during training. Additionally, the GRU network exhibits faster model convergence. Unlike the LSTM network, the GRU network has an update and reset gate. The update gate retains useful details about the input sequences by determining which past information should be passed forward from previous time steps. On the other hand, the reset gate identifies and discards unnecessary information from the network.

The functions of the update gate, *z*_*t*_, and the reset gate, *r*_*t*_, are defined as follows:

$$z_{t}=\sigma (W^{\left(z\right)}x_{t}+U^{\left(z\right)}h_{t-1})$$ (1)

$$r_{t}=\sigma (W^{\left(r\right)}x_{t}+U^{\left(r\right)}h_{t-1})$$ (2)

where *σ*() is the sigmoid function, *x*_*t*_ is the input timestep at time *t* to network, *h*_*t*−1_ is the hidden layer at the previous timestep at time *t*−1, *W*^(*z*)^ and *W*^(*r*)^ are the respective weights of the input timestep, *x*_*t*_, at the update and reset gates, and *U*^(*z*)^ and *U*^(*r*)^ are the respective weights of the hidden layer at the previous timestep, *h*_*t*−1_ at the update and reset gates. The update gate’s equation is the same as the reset gate. The difference between both gates is the associated weights and how the gates are implemented.

Firstly, the candidate hidden state, *h*′_*t*_, is computed using the reset gate to identify and store significant past information. This state is calculated as follows:

$${h\prime }_{t}=\tanh\left(Wx_{t}+r_{t}⨀Uh_{t-1}\right)$$ (3)

where tanh () is a non-linear activation function and *r*_*t*_ is the output of the reset gate. During training, GRU networks learn to assign value to *r*_*t*_ that ranges from zero to one, depending on how much past information needs to be kept. For instance, if *r*_*t*_ is zero, then the past information is completely removed and vice versa.

Finally, the candidate hidden state and the update gate are used to compute a new hidden state, which stores information from the current input timestep and propagates it to the next. The equation to compute the new hidden state is written as follows:

$$h_{t}=z_{t}⨀h_{t-1}+(1-z_{t})⨀{h\prime }_{t}$$ (4)

where *z*_*t*_ is the update gate’s output, and (1−*z*_*t*_) is a term that determines how much current information can be passed on to the next. For example, if *z*_*t*_ is set close to one, the model retains most of the information from the previous hidden layer. On the other hand, if *z*_*t*_ is set close to zero, the model will give precedence to new information. Considering the simplicity and effectiveness of the GRU layers, these components are integrated into the proposed TCN-GRU model.

### 2.3 Classification

Before classifying the generated feature maps from the GRU layers into respective classes, these feature representations are passed into a Global Average Pooling (GAP) layer. Rather than adopting a fully connected dense layer, the GAP layer is employed in this work for its efficiency and simplicity, in contrast to adopting a fully. Unlike the GAP layer, which can adapt different input dimensions, the fully connected dense layer requires input sequences to be flattened to a specific dimension. Furthermore, applying a fully connected dense layer to a network presents several drawbacks. One significant concern is the increase in the overall model parameters, making the model more complex. Consequently, the complex network will be highly vulnerable to overfitting problems.

In contrast, the GAP layer operates in a manner that significantly reduces the overall number of trainable parameters. The GAP layer condenses the feature maps into a compact representation. For example, when processing input with 128 channels of one-by-thirty-two, the GAP layer performs pooling operations to condense the data into 128 channels of one-by-one output. This operation eliminates a large chunk of parameters, reducing the model complexity as well as boosting the training speed. Moreover, the GAP layer does not require parameter optimisation.

Lastly, the computed category confidence maps from the GAP layer are passed to a softmax classifier to classify the feature maps into respective activity classes. A softmax classifier computes the softmax score, *s*_*c*_(*x*) for each class, *c*, using the following equation:

$$s_{c}(x)=x^{T}\theta^{\left(c\right)}$$ (5)

where *x* is the input sequence, *x*^*T*^ is the transposed input sequence and *θ*^(*c*)^ is each class’s dedicated parameter vector. The computed scores are passed into the softmax function, *α*() to estimate the probability of an input sequence, ${\hat{P}}_{c}(x)$, belonging to class *c*. The softmax equation is defined as follows:

$${\hat{P}}_{c}(x)=\alpha {\left(s_{c}(x)\right)}_{c}=\frac{e^{s_{c}(x)}}{\sum_{j=1}^{N}e^{s_{j}(x)}}$$ (6)

where *N* is the number of activity classes. The softmax function is responsible for generating probability for each activity class. The range of the generated probability is between zero and one. The activity class with the highest probability will be predicted as the target class.

Loss functions are immensely significant to deep learning model optimisation during training. These functions measure the classifier’s performance by quantifying the discrepancy between the predicted and true classes. In this study, categorical cross-entropy loss, *CCE*, is adopted to train the proposed TCN-GRU model since the smartphone-based HAR poses a multi-class time series classification challenge. *CCE* computes the loss between the estimated activity class and the true activity class. Then, the calculated error is backpropagated throughout the network to improve the model learning and provide better predictions in the upcoming iteration. The equation of *CCE* loss function is written as follows:

$$CCE=-\sum_{i}^{N}y_{c}^{\left(i\right)}\log\left({{\hat{P}}_{c}(x)}^{\left(i\right)}\right)$$ (7)

where $y_{c}^{\left(i\right)}$ is the ground truth of *i*^*th*^ input sequence belongs to class *c* and ${{\hat{P}}_{c}(x)}^{\left(i\right)}$ is the estimated value of *i*^*th*^ input sequence belongs to class *c*.

## 3 Experimental setups

A clear explanation of the adopted datasets is presented in the following section. Furthermore, the parameter settings of TCN-GRU, as well as the baseline TCN and baseline GRU, are also stated.

### 3.1 Dataset description

In this work, the classification performance of TCN-GRU is validated on two popular publicly available smartphone-based HAR datasets, which are UCI HAR and UniMiB SHAR datasets. The UCI HAR database is a collection of triaxial motion signals from thirty subjects aged 19 to 48, captured by gyroscope and accelerometer sensors embedded in an Android smartphone [17]. During the data collection phase, the volunteers were requested to perform six activities: Walking, Walking Upstairs, Walking Downstairs, Standing, Sitting, and Lying. The smartphone was attached to the volunteers’ waist. The sliding window technique was applied to segment the inertial signals into sample windows of uniform size. On the other hand, the UniMiB SHAR dataset is used for daily activity recognition and fall detection [47]. Thirty volunteers aged 18 to 60 were required to perform seventeen activities: nine activities of daily living (ADL) and eight falls. This dataset involves triaxial inertial signal collection from an accelerometer embedded in an Android smartphone. Table 1 describes each database used in this work.

**Table 1:** Description of UCI HAR and UniMiB SHAR.

Dataset	UCI HAR	UniMiB SHAR
Sensor	Accelerometer and Gyroscope	Accelerometer
Subjects	30	30
Segment Size	128	151
Sampling Rate	50 ms-2	50 ms-2
Channel Size	9	3
Activities (Class Label)	Walking, Upstairs, Downstairs, Sitting, Standing, and Laying	Only the AF-17 set is used with 17 classes: StandingUpFS, StandingUpFL, Walking, Running, GoingUpS, Jumping, GoingDownS, LyingDownFS, SittingDown, FallingForw, FallingRight, FallingBack, HittingObstacle, FallingwithPS, FallingBackSC, Syncope and FallingLeft
Training Testing Split	21 training users: 9 testing users	Five-fold cross-validation (The whole dataset is split into five equally sized sets. In each fold, one set is used for testing and the remaining for training)
Validation Split	10% of the training set	10% of the training set
Validation protocol	Subject Independent Protocol	Subject Dependent Protocol

### 3.2 Model configuration

All the experiments in this work are conducted on a desktop with Intel® Core™ i9-12900K CPU with 2.20 GHz, 32GB RAM, NVIDIA GeForce RTX 3080Ti and 12GB memory. This machine runs on a 64-bit operating system of Windows 10. The proposed TCN-GRU model is developed in Jupyter Notebook, an open-source web-based interactive computing platform, using TensorFlow 2.4.1 and Keras 2.4.0 libraries. Our proposed model is built according to the parameter configuration recorded in Table 2. Furthermore, three models, including baseline TCN 1 (based on the TCN model designed by Lea et al. [34]), baseline TCN 2 (based on the TCN architecture developed by Bai et al. [37]) and baseline GRU network are constructed for performance benchmarking purposes. The parameter settings of baseline TCN 1, baseline TCN 2 and baseline GRU network are presented in Tables 3–5, respectively.

**Table 2:** The parameter settings of the proposed TCN-GRU.

Parameters	UCI HAR	UniMiB SHAR
Input dimension	(128,9)	(151,3)
Batch size	32	32
Number of TCN blocks	1	1
Number of filters	32	32
Filter size	2	3
Dilation rate	1, 2, 4, and 8	1, 2, 4 and 8
Pooling size	4	4
GRU units	128	128
Stride	1	1
Dropout rate	0.05	0.05
Number of epochs	100	100
Initial learning rate	0.001	0.001
Reduce learning on plateau function.	Minimum learning rate = 0.0001	Minimum learning rate = 0.0001
	Mode = validation loss	Mode = validation loss
	Patience = 3	Patience = 3
Optimiser	Adam	Adam
Loss function	Categorical cross-entropy	Categorical cross-entropy

**Table 3:** The parameter settings of the baseline TCN 1.

Parameters	UCI HAR	UniMiB SHAR
Input Dimension	(128,9)	(151,3)
Batch Size	32	32
Number of Blocks	3	3
Number of Filters	32	32
Filter Size	3	3
Dilation Rate	1, 2, 4, and 8	1, 2, 4, and 8
Stride	1	1
Spatial dropout Rate	0.05	0.05
Number of Epoch	100	100
Initial Learning Rate	0.001	0.001
Reduce Learning on Plateau Function.	Minimum learning rate = 0.0001	Minimum learning rate = 0.0001
	Mode = validation loss	Mode = validation loss
	Patience = 3	Patience = 3
Optimiser	Adam	Adam
Loss function	Categorical cross-entropy	Categorical cross-entropy

**Table 4:** The parameter settings of the baseline TCN 2.

Parameters	UCI HAR	UniMiB SHAR
Input Dimension	(128,9)	(151,3)
Batch Size	32	32
Number of Blocks	3	3
Number of Filters	32	32
Filter Size	3	3
Dilation Rate	1, 2, 4, and 8	1, 2, 4, and 8
Stride	1	1
Dropout Rate	0.05	0.05
Number of Epoch	100	100
Initial Learning Rate	0.001	0.001
Reduce Learning on Plateau Function.	Minimum learning rate = 0.0001	Minimum learning rate = 0.0001
	Mode = validation loss	Mode = validation loss
	Patience = 3	Patience = 3
Optimiser	Adam	Adam
Loss function	Categorical cross-entropy	Categorical cross-entropy

**Table 5:** The parameter settings of the baseline GRU network.

Parameters	UCI HAR	UniMiB SHAR
Input Dimension	(128,9)	(151,3)
Batch Size	32	32
Number of GRU layers	4	4
Number of GRU units	128	128
Number of Epoch	100	100
Initial Learning Rate	0.001	0.001
Reduce Learning on Plateau Function	Minimum learning rate = 0.0001	Minimum learning rate = 0.0001
	Mode = validation loss	Mode = validation loss
	Patience = 3	Patience = 3
Optimiser	Adam	Adam
Loss function	Categorical cross-entropy	Categorical cross-entropy

All the models are equipped with their respective hyperparameters and trained on the same training samples for 100 epochs with an initial learning rate of 0.001. The training process is integrated with a dynamic learning rate using the Reduce learning on plateau function for more adaptable and faster model convergence. The ModelCheckpoint function is implemented during model training to store the current optimal model for class prediction. Whenever the model’s validation loss decreases during an epoch, this function stores the model in a folder. This facilitates the selection of an optimal model before it overfits. Lastly, the selected model is tested on unseen test samples.

## 4 Experimental results and discussions

The following subsections discuss the ablation studies, performance evaluations of TCN-GRU architecture, and an analysis of the empirical results.

### 4.1 Ablation study

We conduct an ablation study on the proposed TCN-GRU model to understand how the structural parameters influence classification performance. Seven experiments are conducted on the proposed TCN-GRU using the UCI HAR database: causality, convolutional kernel sizes, number of TCN blocks, bidirectionality of GRU, number of GRU layers, number of GRU units and pooling types. The major challenge in building an effective HAR model is to balance the trade-off between achieving high classification accuracy and maintaining low overall model parameters for computational efficiency. Thus, these experiments aim to identify the optimal hyperparameter values of the proposed model to achieve better classification performance while possessing a relatively low number of trainable parameters.

Causality is crucial in time series classification. In other words, changing the ordering of the time series data may deteriorate the classification performance as the time-dependent information is lost inadvertently. Hence, the effect of causality on the activity classification performance of the proposed TCN-GRU model is analysed by employing two different convolutional layers: 1D dilated causal convolution and 1D dilated convolution. From the results recorded in Table 6, we notice that the model with causality performs better than without causality due to the former’s ability to preserve the order of the input sequence. The causal padding in the 1D dilated causal convolution constricts the direction of information flow throughout the network. Consequently, the model generates a feature map at time *t* solely relying on the input timesteps from *t* and earlier in the preceding layer, disregarding any future timesteps. Therefore, the input sequence’s order is preserved, preventing future information leakage into the past, which is crucial to human motion classification [40, 48]. In contrast, 1D dilated convolution does not restrict the direction of the information flow, and the feature map at time *t* is generated using input sequences from *t* and earlier in the preceding layer as well as those after time *t*. Since causality boosts the model performance, 1D causal convolutions are integrated into the proposed TCN-GRU.

**Table 6:** Effect of causality on the proposed TCN-GRU.

Causality	Precision	Recall	F1 Score	Accuracy (*%*)
1D dilated causal convolution	0.9744	0.9727	0.9730	97.25
1D dilated convolution	0.9667	0.9645	0.9644	96.47

Capturing longer time series is crucial for higher recognition accuracy in time series classification tasks, specifically in the HAR [49]. Indeed, the convolutional kernel’s elongation is a common practice for extracting long temporal features. Despite that, implementing longer kernels can drastically increase the number of parameters, making the model more susceptible to overfitting problems and leading to performance deterioration [49]. Thus, an experiment is conducted to determine the optimal convolutional kernel size that could improve classification performance with minimised parameters. The performance of TCN-GRU with varying convolutional kernel sizes is recorded in Table 7. From the empirical results, we can notice that the proposed model with the convolutional kernel size of 2 achieves the best performance with 97.25% accuracy. This model attains superior performance while possessing the least parameters with only ~0.18 million, substantially less than the other models with longer kernel sizes.

**Table 7:** Performance of the proposed TCN-GRU with different convolutional kernel sizes.

Convolutional kernel sizes	Number of Parameters (*Million*, *M*)	Precision	Recall	F1 Score	Accuracy (*%*)
2	183014	0.9744	0.9727	0.9730	97.25
3	190470	0.9471	0.9427	0.9441	94.40
4	197926	0.9684	0.9655	0.9660	96.57
5	205382	0.9633	0.9618	0.9614	96.17

The TCN block is one of the main constituents of the proposed TCN-GRU model. It is responsible for capturing long temporal dependencies. An experiment is carried out to study how the number of TCN blocks impacts the model accuracy. The general assumption is that if the deep learning model is too shallow, then this model will lose the capability to capture the salient deep features/patterns from the input sequences, causing model underfitting. However, this is not the case in our proposed model. Table 8 shows that the model with one TCN block obtains the highest accuracy and produces the lowest model parameters. Since the proposed TCN-GRU is equipped with dilations, residual connections, and GRU layers, the model can capture the underlying implicit patterns from the input signals. These implicit data patterns retrieve meaningful information about the data. Thus, the model can comprehend the intricate characteristics and dynamics of the inertial motion signals, leading to improved classification performance. On the other hand, it is observed that increasing TCN blocks can cause performance degradation. This implies that the additional blocks do not contribute considerably to the feature extraction. Therefore, the proposed TCN-GRU model employs only one TCN block before the implementation of GRU layers.

**Table 8:** Performance of the proposed TCN-GRU with a different number of TCN blocks.

Number of TCN blocks	Number of Parameters (*Million*, *M*)	Precision	Recall	F1 Score	Accuracy (*%*)
1	183014	0.9744	0.9727	0.9730	97.25
2	204518	0.9710	0.9682	0.9687	96.81
3	226022	0.9652	0.9610	0.9616	96.03
4	247526	0.9685	0.9672	0.9671	96.71

The following experiments are to investigate and manipulate the architecture of GRU layers to identify the optimal GRU structure that improves model performance while maintaining low model parameters, thereby reducing the computational cost. Firstly, the influence of bi-directionality on classification performance is investigated. Implementing bi-directionality in the recurrent model has been validated to be successful in the context of HAR, as it yields satisfactory performances. However, introducing bi-directionality into the proposed TCN-GRU does not improve the model performance. From Table 9, we can see that the uni-directional model outperforms the other bi-directional models. In a bi-directional GRU structure, two separate recurrent layers work simultaneously, and the information flows in two directions (i.e., forward and backward). This allows the model to simultaneously process previous and subsequent information about the input sequences [50]. However, this bidirectional flow violates the network’s causality, yielding suboptimal performance, as depicted in Table 6. Furthermore, the bi-directionality doubles the recurrent settings, making the model expensive to train. As observed from Table 9, there is a noticeable increase in the parameter count between the uni-directional GRU (128 units) and the bi-directional GRU (128 units), where the bi-directional GRU doubles the model parameters of the uni-directional GRU, leading to an increase in training costs [31]. In this study, uni-directional GRU layers with 128 units are employed in the proposed TCN-GRU because these layers demonstrate the highest performance.

**Table 9:** Effects of bi-directionality on the proposed TCN-GRU.

Number of GRU layers	Number of Parameters (*Million*, *M*)	Precision	Recall	F1 Score	Accuracy (*%*)
Uni-directional GRU (128 units)	183014	0.9744	0.9727	0.9730	97.25
Bi-directional GRU with 32 units	52582	0.9584	0.9547	0.9552	95.49
Bi-directional GRU with 64 units	133862	0.9668	0.9653	0.9654	96.57
Bi-directional GRU with 128 units	443878	0.9478	0.9462	0.9458	94.67

Next, the proposed TCN-GRU is tested with different numbers of GRU layers to determine the optimal number of GRU layers. This experiment aims to determine whether increasing the number of GRU layers can impact the information gained on the input signals, leading to higher recognition accuracy. Table 10 records the proposed model’s performance with different GRU layer settings. From the empirical results, we can notice that when the number of GRU layers increases, the overall parameters also increase. However, the model accuracy declines when GRU layers exceed 2. Having a large number of trainable parameters might cause the model to be complex and overfit the training samples, affecting the generalisation capability and model performance. Therefore, the number of GRU layers is set to 2 for the proposed TCN-GRU in this study.

**Table 10:** Performance of the proposed TCN-GRU with a different number of GRU layers.

Number of GRU layers	Number of Parameters (*Million*, *M*)	Precision	Recall	F1 Score	Accuracy (*%*)
1	83430	0.9524	0.9455	0.9456	94.50
2	183014	0.9744	0.9727	0.9730	97.25
3	282598	0.9677	0.9667	0.9668	0.9671
4	382182	0.9405	0.9364	0.9377	93.89

Furthermore, the number of GRU units in the proposed TCN-GRU is manipulated to examine its influence on the overall model performance. This experiment aims to identify the optimal number of GRU units for each layer. Table 11 presents the performance of the proposed TCN-GRU with different numbers of GRU units. We can observe that when the GRU units double, the model parameters grow exponentially. In other words, increasing the number of GRU units escalates the count of trainable parameters, increasing the computational complexity. Besides, it is noticed that the model’s accuracy improves as the GRU units in each layer increase from 32 to 128 units, but the performance deteriorates when the GRU unit is set to 256 units. Hence, we set the number of GRU units at 128 in the proposed TCN-GRU for subsequent experiments.

**Table 11:** Performance of the proposed TCN-GRU with different numbers of GRU units.

Number of GRU units	Number of Parameters (*Million*, *M*)	Precision	Recall	F1 Score	Accuracy (*%*)
32	33446	0.9541	0.9522	0.9524	95.28
64	64870	0.9566	0.9548	0.9550	95.45
128	183014	0.9744	0.9727	0.9730	97.25
256	640486	0.9640	0.9637	0.9645	96.40

As illustrated in Fig 2, there is a pooling layer between a TCN block and GRU layers in the proposed model. This architecture aims to reduce the dimensionality of the extracted feature maps before passing them into the GRU layers. This dimensionality reduction helps diminish the computational load. In this section, an experiment is conducted to assess the need for pooling within the model architecture and which type of pooling method works best for HAR tasks. Table 12 records the performance of the proposed TCN-GRU with different pooling settings: (1) without pooling, (2) with average pooling and (3) with max pooling. From the result, the model with average pooling obtains the lowest classification accuracy. This underwhelming performance may be due to the nature of the pooling function where the feature maps are averaged for downsampling, causing the model to lose the ability to capture the underlying implicit pattern of the input sequence. On the contrary, the model with max pooling achieves the highest accuracy of 97.25%. Owing to this observation, the proposed model adopts a max pooling layer between the TCN block and GRU layers in this study.

**Table 12:** Performance of the proposed TCN-GRU with different pooling settings.

Pooling	Precision	Recall	F1 Score	Accuracy (*%*)
None	0.9623	0.9610	0.9610	96.06
Average pooling	0.9537	0.9520	0.9519	95.22
Max pooling	0.9744	0.9727	0.9730	97.25

### 4.2 Performance evaluation and comparison

Performance analysis between the proposed TCN-GRU, baseline TCN 1, baseline TCN 2, and baseline GRU is presented in this section. Furthermore, the performance comparison of the proposed TCN-GRU and other existing smartphone-based HAR models is also discussed in the section.

#### 4.2.1 UCI HAR

**Fig 6:**
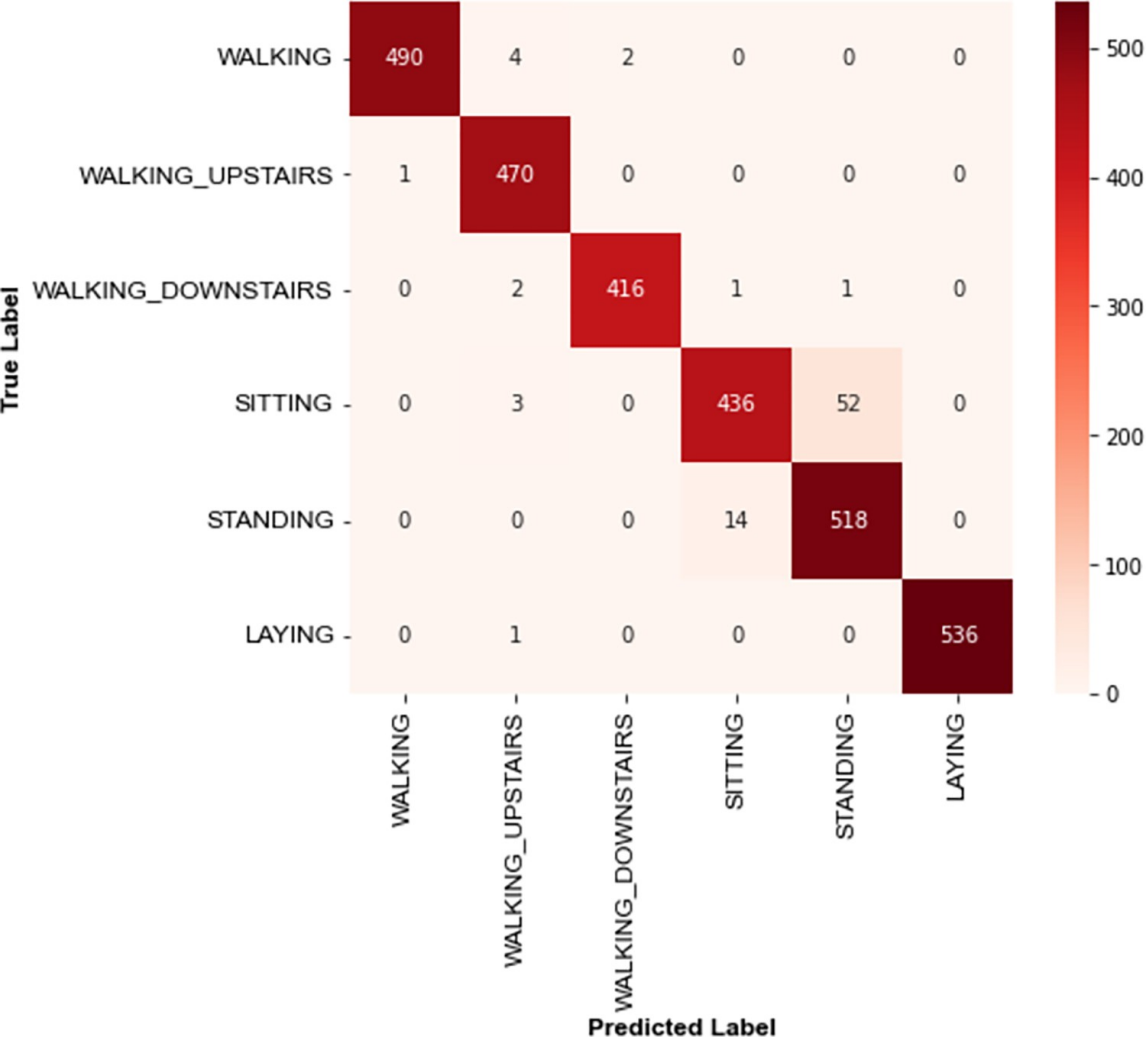
Confusion matrix of the proposed TCN-GRU on UCI HAR.

**Fig 7:**
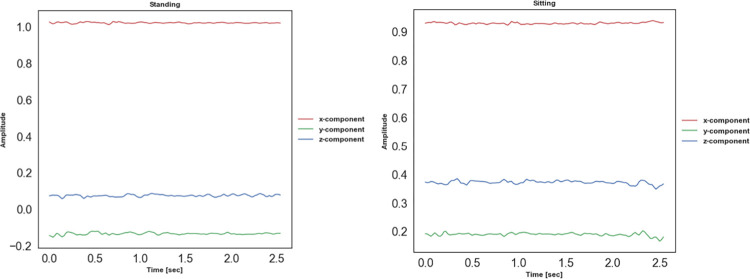
Data visualisation of inertial data signals of the *standing* and *sitting* classes.

Model performance evaluations on UCI HAR are conducted using a user-independent testing protocol. In this protocol, the training set consists of inertial signals from a group of users, and the test set contains inertial signals from the remaining users. To be specific, the training and test set are mutually exclusive. The models are trained on samples from the first twenty-one users and tested with the samples from the remaining users. 10% of the training data is used as a validation subset for model hyperparameter tuning. Fig 6 illustrates the confusion matrix of the TCN-GRU model. It is observed that the proposed TCN-GRU exhibits low misclassifications across all activities except for the *sitting* class. Almost 12% of *sitting* class samples are misclassified as *standing* class. This could be low interclass variance between the inertial data signals of the *sitting* and *standing* classes, as depicted in Fig 7. The similarity in the data patterns between these two activity classes poses a challenge to the model for adequate data differentiation.

**Fig 8:**
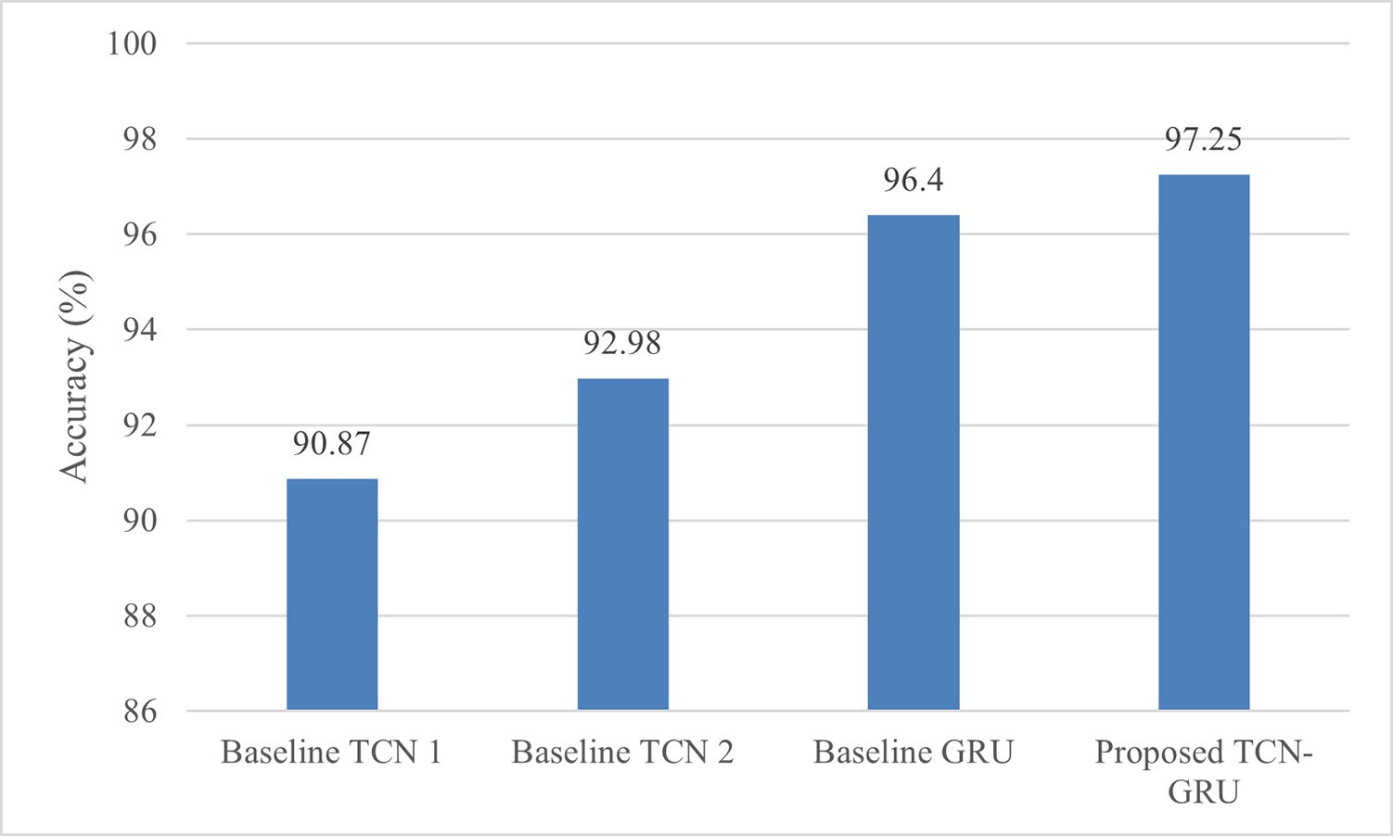
Classification accuracy comparison between the proposed TCN-GRU and the other baseline models on UCI HAR.

Fig 8 shows the accuracy performance of TCN-GRU and the baseline models. As illustrated in the figure, the proposed TCN-GRU outperforms the other baseline models by achieving 97.25% with the least number of parameters. The amalgamation of a TCN model and a GRU network heightens the model’s competence to analyse and capture richer information from the inertial data signals. Typically, with a small training sample size, deep learning classifiers usually tend to overfit the training data, compromising the models’ capability to generalise to unseen data. Considering the promising performance coupled with relatively low model parameters, it is reasonable to infer that the proposed model is not too complex and is less susceptible to overfitting problems. Compared with the other baseline models, the baseline GRU obtains the highest accuracy score of 96.40%. This performance deduces that the GRU network is more effective at extracting significant temporal dependencies from the input sequences. The recurrent nature and gating mechanisms in the GRU enable it to retain and update appropriate information over time, making it effective in comprehending the temporal dependencies in the data [51, 52]. However, compared with the proposed TCN-GRU model, our model exhibits advantages by possessing the lowest model parameters and requiring the shortest training time.

Table 13 records the accuracy comparison between the proposed TCN-GRU and the other existing HAR models. Generally, the proposed TCN-GRU outperforms the other existing HAR models, including handcrafted feature-based and deep learning methods, with an accuracy score of 97.25%. Specifically, the proposed architecture dominates the handcrafted feature-based methods by 1% to 14%. Handcrafted feature-based methods such as RF, KNN, and RK-KNN rely on manual feature engineering techniques, which compute features from input sequences based on prior knowledge. These algorithms are suboptimal in effectively generalising unseen test data, leading to performance deterioration. On the other hand, the proposed TCN-GRU automatically captures longer temporal dependencies from the input sequences. By amalgamating the TCN and GRU components, the proposed model can comprehend the intricate dynamic patterns in the data, leading to improved prediction performance. Although the proposed model outperforms the other deep learning models, the difference in classification accuracy between the proposed model and several deep learning models (i.e. CNN with Lego filters, ResNet with heterogenous convolution, GRU-INC and Multi Input CNN-GRU) is relatively small, approximately 1%. However, the proposed TCN-GRU possesses a significant advantage over these models, which is lower model complexity. The existing models, such as ResNet with heterogenous convolution, GRU-INC and CNN with Lego filters models, are more complex with gigantic trainable parameters, thereby escalating computational costs. Hence, we can conclude that the proposed TCN-GRU is performing better than the existing models on the UCI HAR dataset in terms of both classification performance and model complexity.

**Table 13:** Performance comparison between the proposed TCN-GRU and the other existing models on UCI HAR.

Methods	Accuracy (*%*)	The Number of Trainable Parameters (Million)
SVM [17]	96.00*	-
RF [19]	83.30*	-
KNN [21]	90.46*	-
RK-KNN [22]	92.67*	-
CNN with Lego filters [5]	96.90	1.30*
Attention induced multi-head CNN [4]	95.38*	1.51*
ResNet with heterogenous convolution [53]	97.01*	0.42*
Stacked LSTM [8]	93.13*	-
Bidirectional LSTM [31]	92.67*	-
Deep Residual Bidirectional LSTM [7]	93.60*	-
Dilated TCN [10]	93.80*	0.15*
Encoder–Decoder TCN [10]	94.60*	0.16*
InnoHAR [15]	94.50*	
CNN-LSTM [14]	92.13*	-
iSPLInception [54]	95.09*	1.33*
Multi input CNN-GRU [51]	96.20*	-
GRU-INC [55]	96.27	0.67*
Baseline TCN 1	90.87	1.73
Baseline TCN 2	92.98	0.28
Baseline GRU	96.40	0.35
Proposed TCN-GRU	97.25	0.18

* Results extracted from the respective articles.

#### 4.2.2 UniMiB SHAR

**Fig 9:**
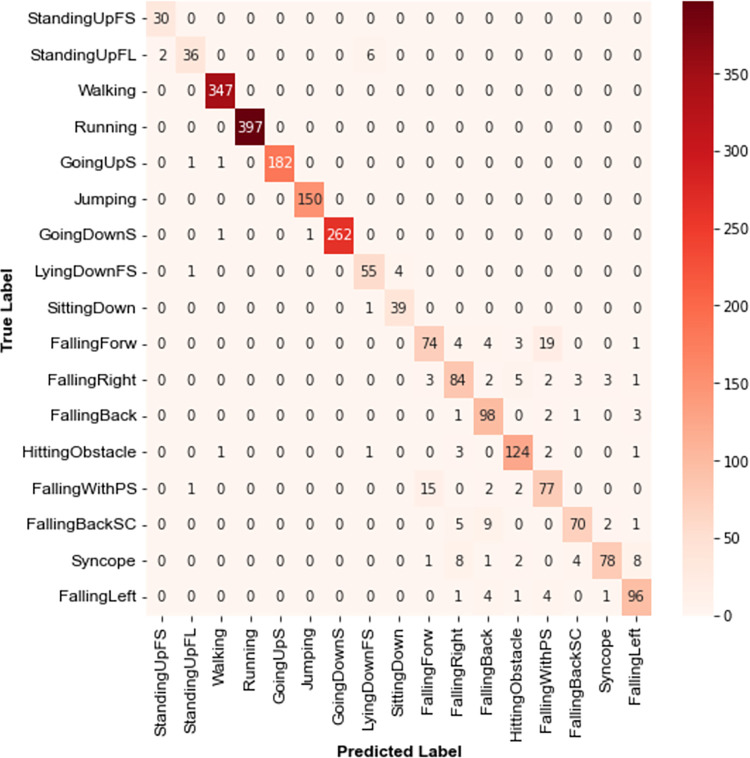
Confusion matrix of the proposed TCN-GRU on UniMiB SHAR.

A subject-dependent testing protocol is applied to the UniMiB SHAR database where the training and test sets contain inertial samples from the same volunteers, but there are no overlapping samples between the sets. The recognition performance of TCN-GRU is validated using a five-fold cross-validation technique. UniMiB SHAR database is split into five equally-sized subsets. During each fold, one subset is used as a testing set, and the remaining subsets are used for model training. A validation set is created from 10% of training samples for each fold. Fig 9 illustrates the confusion matrix of TCN-GRU. The model demonstrates encouraging performance in classifying human activity.

**Fig 10:**
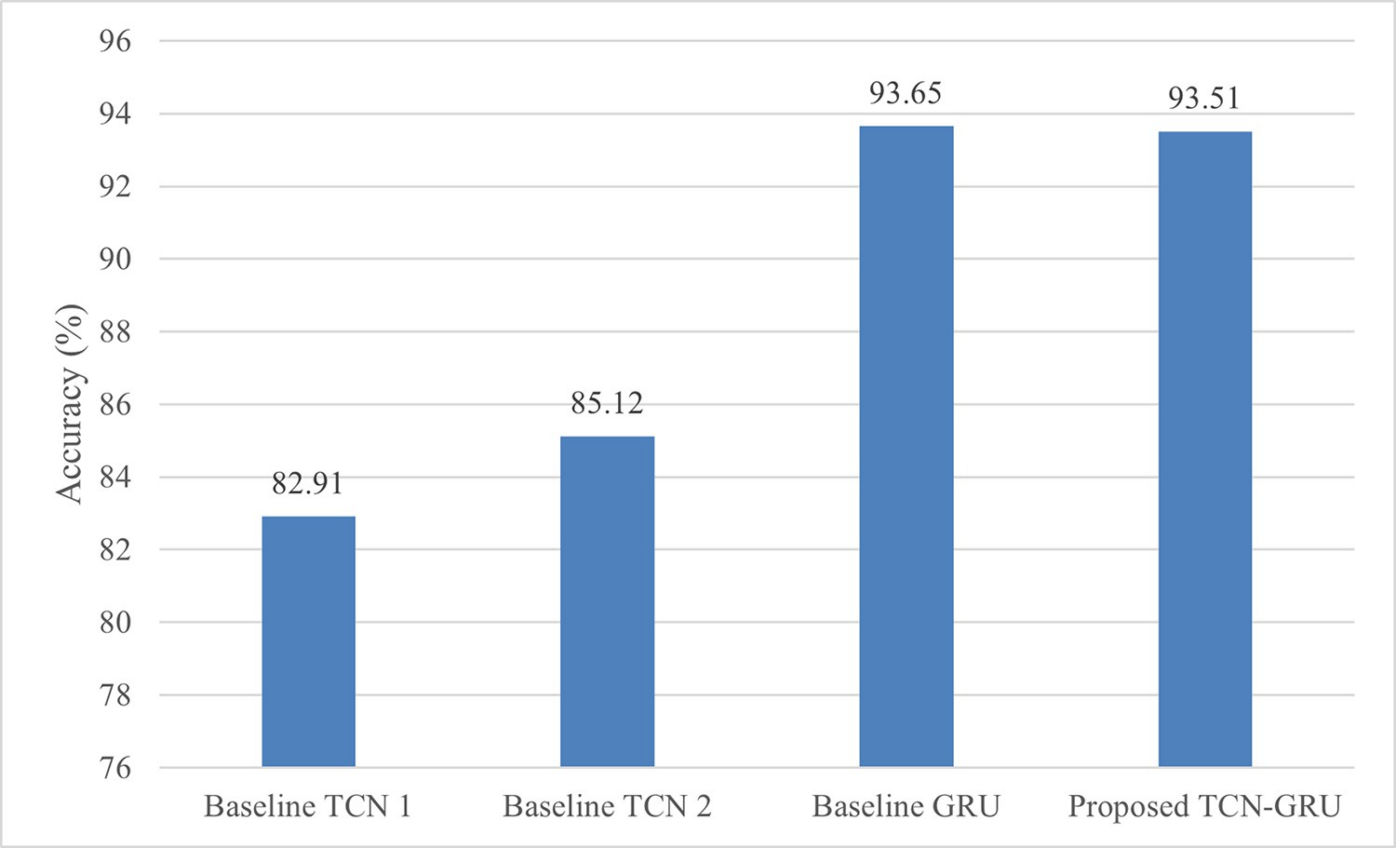
Classification accuracy comparison between the proposed TCN-GRU and other baseline models on UniMiB SHAR.

**Fig 11:**
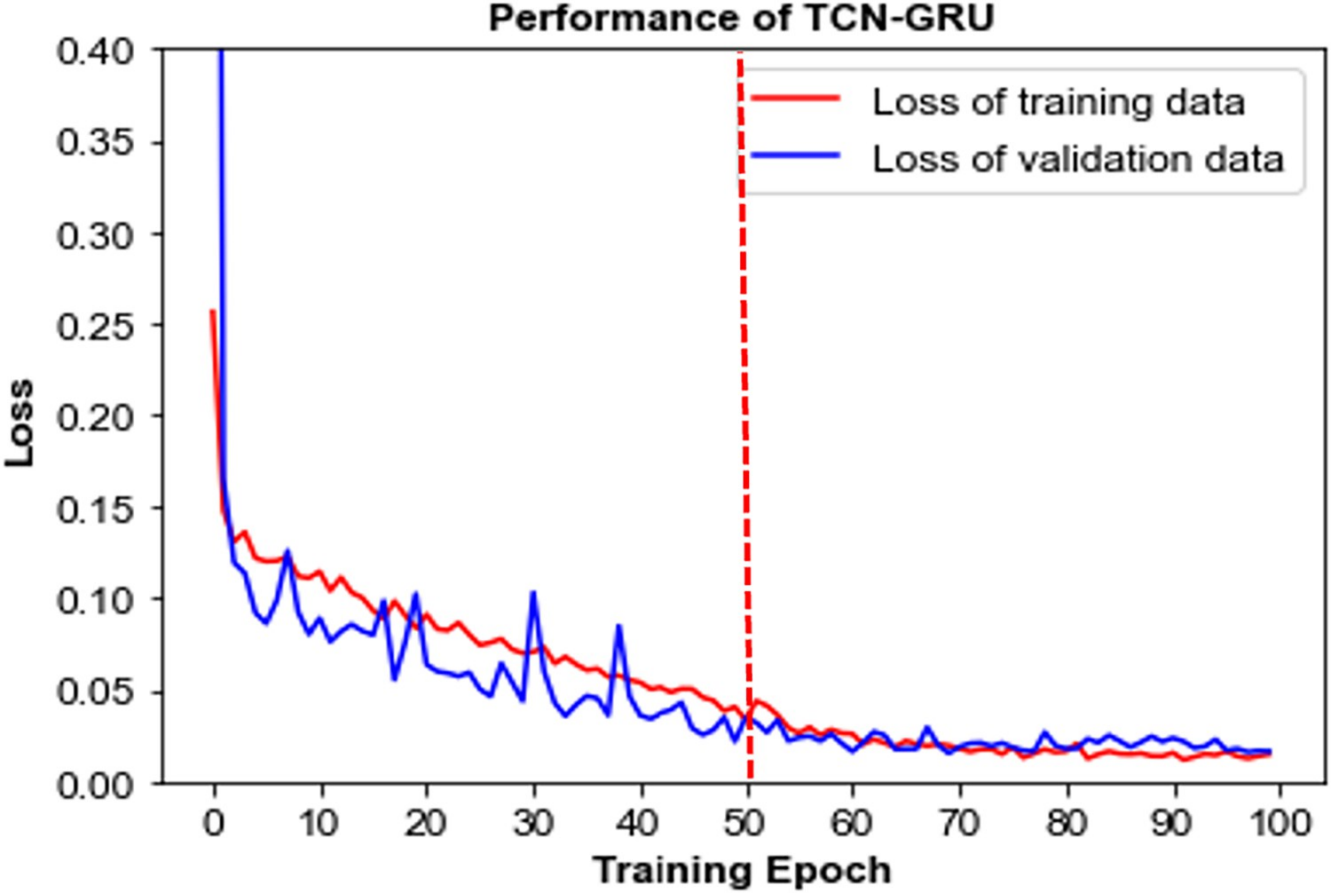
The model convergence of the proposed TCN-GRU.

The accuracy performance of the proposed TCN-GRU and the baseline models is depicted in Fig 10. The experimental result reveals that the proposed model and the baseline GRU model exhibit comparable performance, achieving an accuracy score of approximately 93%, whereas both baseline TCN models attain moderate classification performances with 82.91% and 85.12% accuracy, respectively. It is worth noting that our proposed model achieves such good performance with only half the trainable parameters, i.e. 0.19 million parameters, compared to the baseline GRU model. Furthermore, the proposed TCN-GRU exhibits faster convergence, converging at epoch 50, as illustrated in Fig 11. The fast convergence implies the model’s proficiency in learning intrinsic data patterns, thereby achieving optimal performance while requiring fewer epochs.

Table 14 records the recognition performance of the proposed TCN-GRU and the existing smartphone-based HAR models, including handcrafted feature-based and deep learning methods. The empirical results show that the recognition performance of the proposed TCN-GRU model prevails over the existing models. There is an accuracy difference ranging from 7% to 18% between the proposed model and the other methods, except for the LSTM-XGB model. Moreover, some models, such as CNN+C3 and DanHAR, are indeed deep and complex, with more than 1.5 million trainable parameters. These architectures are burdened by heavy computational load. Even though the Predsim ResNet model has the lowest number of model parameters, with only 0.11 million, it achieves only 80.33% accuracy.

**Table 14:** Performance comparison between the proposed TCN-GRU and the other existing models on UniMiB SHAR.

Methods	Accuracy (*%*)	The Number of Trainable Parameters (Million)
KNN [47]	82.86*	-
Ensemble subspace KNN [57]	86.00*	-
HS-CNN [58]	77.26*	0.51*
HS-ResNet [58]	79.19*	0.72*
Asymmetric Residual Neural Network [59]	76.39*	-
Predsim [60]	78.07*	0.55*
CNN-TAMA [61]	77.01*	0.35*
CNN+C3 [62]	75.16*	1.52*
DanHAR [63]	79.03*	2.40*
Selective Kernel CNN [64]	76.84*	0.54*
Predsim ResNet [65]	80.33*	0.11*
LSTM-XGB [56]	92.59*	-
Baseline TCN 1	82.91	1.02
Baseline TCN 2	85.12	0.39
Baseline GRU	93.65	0.35
Proposed TCN-GRU	93.51	0.19

* Results extracted from the respective articles.

On the other hand, the LSTM-XGB model proposed by Mekruksavanich et al. [56] demonstrates promising classification performance with 92.59% accuracy. Yet, implementing the LSTM network increases model complexity and escalates computational costs during model training. In contrast, our proposed model tackles this issue by integrating TCN and GRU models. Both TCN and GRU models are computationally lightweight, ensuring efficient processing. Furthermore, the amalgamation allows the proposed architecture to acquire extended temporal dependencies from inertial signals and retain them throughout the network, mitigating the vanishing and exploding gradient effects. Consequently, the proposed TCN-GRU attains remarkable classification performance with minimal trainable parameters.

## 5 Conclusion

A lightweight deep learning model that amalgamates a TCN model with a GRU model, coined TCN-GRU, is proposed for smartphone-based human activity recognition. With only 0.18 million trainable parameters, this architecture supports automatic spatiotemporal feature learning from the input inertial signals without tedious manual feature engineering. The overall model complexity is reduced by implementing dilations and smaller filters, which inherently reduces the model parameters. Consequently, the proposed model becomes less prone to overfitting. Human activity recognition is a time series classification problem where the sequential order of inertial data is significant. The dilated convolutions in the proposed model are embedded with causal padding to preserve the temporal order of the input sequence. Causal padding ensures that the information flow is strictly from the past to the future and prevents future information from leaking into the past. Another significant contribution of the proposed TCN-GRU is its capability to extract and preserve long temporal features from the input sequences. These features provide richer information to the model for better data analysis. In addition, incorporating the residual connections and GRU layers in the proposed TCN-GRU model alleviates the vanishing and exploding gradient effects, ensuring stable gradient flow throughout the network. The feasibility of the proposed TCN-GRU in classifying human activity is validated on UCI HAR and UniMiB SHAR datasets. The empirical results denote that the proposed TCN-GRU achieves promising classification performances on both databases with lesser trainable parameters by attaining accuracies of 97.26% on UCI HAR and 93.51% on UniMiB SHAR. The empirical results exhibit that similar inertial signals cause low interclass variances, leading to the misclassification of data. In our future work, appropriate data engineering approaches such as discriminant data transformation will be explored to maximise the interclass variance, enhancing classification performance. Additionally, the application of human activity recognition has potential negative societal implications, primarily concerning data privacy. This behaviour data collected through HAR systems could reveal information about individuals’ health conditions or other personal matters. Thus, future work will also explore federated learning for decentralised data processing to minimise the exposure of sensitive information by giving users more control over their data. Achieving a balance between data privacy protection and recognition performance will be prioritised during the development of federated learning for human activity recognition.
